# Roles of Extension Officers to Promote Social Capital in Japanese Agricultural Communities

**DOI:** 10.1371/journal.pone.0091975

**Published:** 2014-03-18

**Authors:** Kosuke Takemura, Yukiko Uchida, Sakiko Yoshikawa

**Affiliations:** 1 Graduate School of Management, Kyoto University, Kyoto, Japan; 2 Kokoro Research Center, Kyoto University, Kyoto, Japan; Mälardalen University, Sweden

## Abstract

Social capital has been found to be correlated with community welfare, but it is not easy to build and maintain it. The purpose of the current study is to investigate the role of professional coordinators of social relationships to create and maintain social capital in a community. We focused on extension officers in Japanese agricultural communities, who help farmers in both technical and social matters. A large nation-wide survey of extension officers as well as two supplementary surveys were conducted. We found that (1) social capital-related activities (e.g., assistance for building organizations among farmers) were particularly effective for solving problems; (2) social capital (trust relationships) among community residents increased their life quality; (3) social capital in local communities was correlated with extension officers' own communication skills and harmonious relationships among their colleagues. In sum, social capital in local communities is maintained by coordinators with professional social skills.

## Introduction

As humans live in societies, they are more or less social and interwoven in the society [1]. Given that humans significantly rely on social relationships to survive [2], mutual cooperation within community is essential. However, a state of mutual cooperation is not necessarily easy to establish. Tragedy of the commons, which was initially proposed by Hardin [3], is a typical example that highlights the conflicting nature of individual self-interests versus public goods. It has been suggested that one of the key factors to deal with this difficulty of social life and establish mutual cooperation efficiently is social capital [4]. The purpose of the current research is to investigate consequences (e.g., social capital brings improvement of the living condition in a community) as well as antecedents (e.g., personal social skills or positive social interactions with others) of social capital, by focusing on roles of professional social coordinators in agricultural communities.

### Social capital

The term social capital captures the idea that social bonds and norms are important for people and communities [4]–[5]. Social capital can be broadly defined as the benefits of investing in social relationships [6], similar to financial capital and human capital (investing in individual capacities such as education). An extensive literature shows that human welfare depends heavily on social capital and also that social capital varies widely among human social environments. A recent study by Gutiérrez, Hilborn, and Defeo [7] have found, for instance, that social capital was a contributing factor in preventing the tragedy of the commons and leaded to the success of community-based co-management fisheries. Also, Sampson, Raudenbush, and Earls [8] showed that neighborhood-level collective efficacy, comprised of social capital and informal social control, reduced violent acts such as homicide. Social capital has also been suggested to increase regional incomes, life satisfaction, and life expectancy (see [4], [9], [10], for reviews).

Why does social capital or social cohesion improve the quality of life in the community? For example, a trust relationship, one feature of social capital [4], [9], enhances cooperation, and so reduces transaction costs between people. Instead of having to invest in monitoring others, individuals are able to trust them to act as expected, thus saving money and time [4]. Accordingly, it becomes possible for them to invest money and time in other things such as health (see also [11]–[12], for reviews on how social relationships promote physical health). Also, mutual cooperation that social capital promotes is essential to build and/or maintain public goods such as irrigation systems in agricultural communities and sustainable resources [7], [13].

Although it is becoming well-known and accepted that social capital is important for people and communities, it is not easy to build and maintain it. Imagine someone has altruistic intention and is willing to have reciprocal relationships with others. Even in this instance, it is not always easy to promote reciprocal relationships because his or her intention is not transparent to others. He or she needs to prove his or her good will so that others feel safe to step into the relationship. Norms, which are another important component of social capital that help establish mutual cooperation [9], generally take time to be established. Sampson et al. [8] have found that residential instability is negatively associated with neighborhood-level collective efficacy, which, as mentioned above, comprised mutual control against norm violation. Thus, in neighborhoods where residents change frequently, collective efficacy tends to be low and consequently violence tends to be more prevalent. Moreover, even if mutual cooperation is established, maintaining it is not easy. It is generally observed in public goods game experiments that cooperation decreases as the game proceeds [14]–[15]. Trust relationships, which promote cooperation, are also very sensitive and easy to be broken. Once installing a monitoring and sanctioning system against free-riders, group members' trust ironically becomes even lower than initial levels after removing the sanctioning system [16].

### Role of the intermediary

The current paper examines roles of individuals playing as an *intermediary* or *coordinator* of social relationships, which has been suggested to create and maintain social capital. For example, it has been suggested that the existence of an intermediary promotes the building of trust relationships [17]. Suppose that one person needs to decide whether he or she trusts another person whom he or she has just met. In this situation, trust tends to be promoted if there is someone whom both the trustor and trustee have already established relationships with. The first reason is that uncertainty for the trustor about the trustee's personality is reduced through confidence in the intermediary as a good judge of people. Second, the existence of the third person changes incentive structures for the trustee. If the trustee cheats the trustor, the relationship between the trustor and the intermediary may be damaged. Thus, cheating the trustor will bring negative consequences not only to the trustor, but also to the intermediary. As a result, the relationship between the trustee and the intermediary will also be potentially damaged by the trustee's ill-intended behavior against the trustor. Knowing such potential consequences, the trustee has no (or at least smaller) incentive to cheat the trustor. Given this incentive structure, the trustor now feels secure to trust this person (there is *assurance* in Yamagishi's [18] terminology). After all, the existence of an intermediary promotes the building of trust between two parties who have no established relationship. These mechanisms pointed out by Coleman [17] are examples of how intermediaries serve in constructing social capital.

In line with Coleman's argument, Harada et al. [19] found that public health nurses, professionals that support community residents' health, played the role of intermediary in social relationships. They suggested that public health nurses helped to strengthen relationships within local communities (i.e., bonding social capital; [9]), and contributed to establishing relationships between community residents and the government as well as other types of professionals such as nursery school teachers (i.e., bridging social capital; [9]). Though an untrained third person, such as the mutual acquaintance in Coleman's [17] argument, may help social ties to be formed, the findings of Harada et al. [19] suggests that the existence of professionals who have skills for construction and maintenance of social capital is an important key for community welfare. However, Harada et al.'s [19] study, which employed a semi-structured interview method, was limited because of sample size (only 20 public health nurses). Further investigations about roles of professional intermediaries are undoubtedly needed. To this end, the current study examined roles played by a different professional coordinator of social relationships: extension officers working in an agricultural community.

### Extension officers in Japanese agricultural communities

According to Zakaria and Nagata [20], since the end of World War II, Japan started to embark on a concerted effort to revitalize its agriculture sector in order to boost production to meet the escalating demand for food. The central and prefectural governments worked closely to enhance the training of farmers to promote their technical and managerial skills and to ensure sustainability, and this was carried out through the activities and programs by the agricultural extension services.

The Japanese extension system for agriculture, which started in 1948, was meant to help farmers acquire useful, appropriate, and practical knowledge in the domain of agriculture. This system was adapted from the American extension system but modified when applying it in Japan, so that the extension works could well function in Japanese culture for local needs and requirements [21].

As of 2010, there were approximately 7,000 officers in Japan, each of them belonging to a prefectural government. To become an extension officer, one has to pass a national exam. The job of extension officers is to help farmers in person to acquire technical and managerial knowledge and other skills in the domain of agriculture [22]. They do this job in collaboration with the Ministry of Agriculture, Forestry and Fisheries, research institutions, agricultural universities, and the prefectural government.

The Japanese Ministry of Agriculture, Forestry and Fisheries officially states that extension officers are expected to serve two functions: 1) “specialist” function, and 2) “coordinator” function [23]–[24]. Specialist function means “extension activities to provide farmers with advanced techniques and related knowledge (including managerial knowledge and skills), according as appropriate to local environments.” On the other hand, coordinator function means to “help local farmers and related parties share future goals, clarify tasks they need to address, develop an approach to the tasks, and conduct it, under the cooperation with leading farmers as well as relevant organizations within and around local communities.” Thus, extension officers are supposed to help farmers not only with skills and knowledge directly related to agriculture, but also help farmers build and maintain bonding-type social capital (social capitals within local communities) as well as bridging-type social capital (connections with related parties outside of communities).

In fact, Zakaria and Nagata [20] pointed out that extension officers in Japan “as intermediaries and catalysts, are the key links between farmers and the relevant agencies in terms of providing personalized and need-based information for decision making by all parties concerned” (in abstract [20]). Also, “the agricultural extension organizations naturally provide the place or the Japanese concept of ‘ba’ [25], which means 'a shared space that serves as a foundation for knowledge creation' for the promotion of active interactions, consultations and exchanges between extension and farmers” (p. 34 [20]). Also, Fukuda [26], [27] points out that one of the roles extension officers play in Japanese agricultural communities is to facilitate communications and interactions among farmers as well as between farmers and related agencies in order to help innovate techniques and spread knowledge. These arguments suggest the significance of social ties within and around agricultural communities, and roles played by extension officers to construct and maintain them.

### Importance of social capital in agricultural communities

Prior research has suggested that residents of agricultural communities are particularly interdependent and rely on social relationships compared to communities engaged in other types of economic activities such as herding [28]. This implies that social capital is highly important for agricultural communities. Indeed, there are several studies showing the association between social capital and collective actions as well as welfare of agricultural communities. Fukushima et al. [29], for example, found that trust toward other residents in the same local community was positively related with participation in collective management of local resources such as irrigation systems and commons (see also [30], for similar findings in Thailand). Also, trust relationships within agricultural communities have been found to be associated with residents' self-rated health [31]–[32] as well as settlement in the communities [33]. It was also shown that damages by animals, such as monkeys, which brought serious harms to agricultural communities, could be efficiently mitigated by routinely employing community level cooperation to scare away the animals [34]. More generally, Gyawali, Fraser, Bukenya, and Banerjee [35] found that increased human well-being (composite of income, education, and employment) was associated with social capital, through a survey conducted in the west-central Black Belt region of Alabama, which contains vast amounts of forest resources and fertile agricultural land. As a whole, in line with the social capital literature, social capital such as trust relationships have been found to relate to human welfare in agricultural communities.

There are at least two issues awaiting empirical study. First, though it has been suggested that social capital is associated with human welfare in agricultural communities, much of the prior research did not prove that the observed associations are causal. The current study addressed this by analyzing panel data collected through multiple surveys targeting extension officers in agricultural communities (Analyses 1 and 2). Second, we explored what kind of characteristics and situations the extension officers should possess for building social capital efficiently, by analyzing large survey data of extension officers all over Japan (Analysis 3).

### Overview

We conducted three survey studies, and performed several analyses by combining the datasets when necessary. Data 1 constituted our major dataset, which was created by a nation-wide survey for more than 4,000 extension officers all over Japan, conducted in 2010. Data 2 and 3 were studies that included responses of the extension officers in two different areas (Data 2: Kinki area in 2009 and Data 3: Aichi prefecture in 2011). In Data 2 and Data 3, we could identify those who were also included in Data 1 in 2010 and thus we can examine that data as a panel data for a time-series analysis.

We conducted analyses mainly based on Data 1, but used combined datasets as well, depending on the purpose of the analysis. Analyses 1 and 2 addressed the effect of social capital in communities, such as trust relationships among community residents. In Analysis 1 with Data 1, we investigated the effect of extension service activities related to establishing social capital for solving farmers' problems. Though previous studies on extension officers in Japan [20] and the literature on the importance of social capital suggest the significant roles of the officers' coordinator function, this is not necessarily a widely shared notion even among the officers themselves [36], and thus needs to be examined empirically. Analysis 1 compared the performance of extension activities related to social capital and the other types of activities. In Analysis 2, we examined whether trust relationships among community residents (one of the important components of social capital) would increase life quality or not through a time-series analysis based on combined datasets of Data 1, 2, and 3.

In Analysis 3, we investigated what kinds of extension officers were more likely to contribute to trust relationships among community residents. We analyzed Data 1 to examine effects of personal traits of extension officers such as communication skills and the social relationships they had.

We first describe the three datasets we used. Then, we report the results of our Analyses 1 to 3.

## Method

### Ethics statement

The survey for Data 1 was approved by the Japan Agricultural Development and Extension Personnel Association. The survey for Data 2 was approved by the Kinki Regional Agricultural Administration Office. The survey for Data 3 was approved by the Aichi Agricultural Development and Extension Personnel Association. All participants gave consent by completing the survey. Data files are not available due to the consent agreement with the organization of extension officers. Upon request, however, we could provide the detailed information about the data.

### Data 1

#### Respondents

With the cooperation of the Japan Agricultural Development and Extension Personnel Association, we called all the extension officers in Japan (*N* = 7,241) for the study, and 4,355 extension officers participated in the study (response ratio was 60.0%). Respondents from all but two prefectures completed the online survey (for Saitama and Wakayama prefectures, the surveys were sent and returned through the mail because computers at their workplaces could not access the survey website due to access limitations). Data collection was conducted between September and October 2010. Table 1 provides information on gender, age, and years of working experience as extension officers of respondents in Data 1.

**Table 1:** Summary of respondent characteristics.

		Data 1	Data 2	Data 3^a^
Study period		September-October, 2010	July-August, 2009	October, 2011
Population		Extension officer in Japan	Extension officer in Kinki area (6 prefectures)	Extension officer in Aichi prefecture
Sample size		4,355	319	101
Response rate		60%	52%	54%
Gender	Female	23%	18%	68%
	Male	60%	63%	26%
	No response	17%	19%	6%
Age	20s	6%	3%	6%
	30s	18%	18%	16%
	40s	37%	35%	61%
	50s	24%	24%	16%
	60s	1%	2%	0%
	No response	13%	18%	0%
Years of experience working for the current job	3 or less	11%	6%	16%
	Between 4–10	16%	15%	16%
	Between 10–15	15%	17%	6%
	15 or longer	38%	44%	58%
	No response	20%	18%	3%

a Data 3 was planned to be merged with Data 1. To reduce the burden of respondents, we did not ask respondents in Data 3 on their gender, age, or years of working experience. For Data 3, we report information on these variables based on 31 respondents who were identified in Data 1.

#### Measures. Extension activities they had conducted

The respondents were asked to recall one of their recent experiences in which they were faced with a difficulty in the case they were charged with. Then they were instructed to check all the extension activities they had conducted in that agricultural situation from the list of 11 types of activities, which were derived from a previous study [37]: 1) Assistance to foster the sustainable workforce, 2) Assistance to establish the desirable area of productions, 3) Assistance to conduct eco-friendly agriculture, 4) Assistance regarding food safety, 5) Assistance for the development of agricultural communities, 6) Introduction of agricultural techniques, 7) Assistance for sales promotion, 8) Collaboration and coordination with relevant organizations, 9) Assistance for building organizations and collaboration among farmers, 10) Providing a vision for the future, and 11) Identifying specific problems the community has.

After indicating all the conducted activities, they completed the following four items, all of which were considered to reflect their assessment of their activities; the first two items asked how satisfied they were, and how satisfied they thought the community residents were with their activities as a whole in that situation. For both items, they indicated the level of satisfaction using the same scale ranging from 0 to 100. The other two questions were about positive feedbacks from the community residents. The first item asked how often the community residents showed their gratitude to the respondents (from 0 =  Never to 3 =  Very often). The second one asked how pleased the community residents were about the respondent's activities in total (from -3  =  Not pleased at all to 3 =  Fairly pleased). To give equal weights to the four items, we rescaled the items so that each ranged from 0 to 1. The average of the rescaled items forms our measure of performance (α = .87).

#### Perceived state of the community

Another part of the survey was about the state of the community where the respondents were working at that time. The respondents were instructed to indicate life quality of the community residents (2 items, “The community residents are satisfied with their circumstances of life,” “The living conditions of the community residents were all right”; α = .78). The second set of this part was about trust relationships within community (7 items, e.g., “I think the community residents trust each other,” “The interpersonal relationships among the community residents are generally smooth”; α = .82). Response options were provided on 7-point scales (1 =  Strongly disagree to 7 =  Strongly agree).

#### The respondent's skills and social relationships

We administered a modified version of Tsutsui's [38] collaboration activity scale, which was designed to measure a behavioral tendency for collaborations and communications with other related parties such as agricultural cooperatives and local government. Sample items included “I hear about services and actual situations of other related organizations (including resident organizations) from themselves,” “I know what kind of professionals are in other related organizations (including resident organizations),” “I ask for cooperation of other related organizations (including resident organizations),” (α = .84). Response options were provided on 4-point scales (1 =  Not at all to 4 =  Very much). We call this the collaboration index hereafter.

We also asked about their self-evaluation of communication skills (“What do you feel about your own current communication skills as an extension officer?”) and self-evaluation of their knowledge and technical skills (“What do you feel about the current level of your knowledge and techniques which are directly related to extension activities?”), which related to the major functions of extension officers (“coordinator” function and “specialist” function, respectively). For both items, response options were provided on 7-point scales (-3 =  Far from enough to 3 =  Good enough). They also worked on a 10-item scale of extraversion developed by Goldberg [39] including sample items such as “I am the life of the party” and “I start conversations” (1 =  Strongly disagree to 7 =  Strongly agree; α = .91). Extraversion was measured in this study as one of the control variables of personality traits that might promote their coordinator function.

Two questions were administered to assess social relationships the respondents had. The first one was about their relationships with the community they were working for (hereafter referred to as “tie with community”), ranged from -3 (I am independent and separate from the community) to 3 (I am connected with the community). The second one was about relationship harmony at their workplace (i.e., extension center) (-3 =  Very bad to 3 =  Very good).

### Data 2

#### Respondents

With the cooperation of the Research Group of Specialists in Extension Activities of Kinki, we called all the extension officers in six prefectures of the Kinki area (*N* = 616), and 319 extension officers participated in this study (response ratio was 51.8%). The URL of the survey website was announced and the Excel form of the questionnaire was also sent to them via email just in case they could not work on the survey online. Data collection was conducted between July and August, 2009, approximately one year before Data 1.

For both Data 1 and 2, respondents were asked to create a unique identification code for themselves. Out of the 319 respondents of Data 2, 61 people were identified in Data 1 as well, and among them, 29 people had worked for the same community between the two data collections, and completed all the relevant scales.

#### Measures

To measure the perceived trust relationships among the community residents as well as the perceived life quality of the community residents, the identical measures to those of Data 1 were used for Data 2 (αs  = .83 and .76 for trust relationships and life quality of the community residents, respectively). The respondents in Data 2 also indicated how long they had been working for the same community. This information helped us to select the respondents who worked for the same community in Data 1 and 2.

### Data 3

#### Respondents

With the cooperation of the Aichi Agricultural Development and Extension Personnel Association, we called all the extension officers in Aichi prefecture (*N* = 188) for the study, and 101 of them participated in the study (response ratio was 53.7%). The URL of the survey website was announced to all the extension officers in Aichi. They worked on the survey during October 2011, approximately one year after Data 1.

Respondents of Data 3 were asked to create a unique identification code based on the same rule from Data 1 and Data 2. Out of the 101 respondents of Data 3, 47 people were found to be identical in Data 1 as well, and 31 of them had worked for the same community between the two data collections, completed all the relevant scales, and did not learn about the results of Data 1.

#### Measures

Just like Data 2, the same measures for the perceived trust relationships among the community residents and the perceived life quality of the community residents were used for Data 3 (αs  = .77 and .73, respectively). Also the respondents in Data 3 indicated how long they had been working for the same community so that we could select the respondents who worked for the same community in Data 1 and 3.

## Results

### Analysis 1: Types of extension activities and their effects

In Analysis 1, we analyzed Data 1 to see what kind of extension activities were efficient to solve problems that farmers faced. We predict that activities related to social capital (e.g., activities promoting coordination among farmers) are effective to solve the problems among farming communities.

As aforementioned, respondents of Data 1 were asked to recall one of their recent experiences in which they had faced a difficulty, and indicate all the extension activities they conducted in the situation. The most frequently conducted activity was collaboration and coordination with relevant organizations (63%), followed by introduction of agricultural techniques (61%; see Table 2 for the other extension activities).

**Table 2:** Extension activities and implementation rate in difficult situations respondents experienced.

Extension activities	Implementation rate
Assistance to foster the sustainable workforce	50%
Assistance to establish the desirable area of productions	44%
Assistance to conduct eco-friendly agriculture	24%
Assistance regarding food safety	24%
Assistance for the development of agricultural communities	31%
Introduction of agricultural techniques	61%
Assistance for sales promotion	26%
Collaboration and coordination with relevant organizations	63%
Assistance for building organizations and collaboration among farmers	44%
Providing a vision for the future	36%
Identifying specific problems the community has	38%

What kinds of activities have a positive effect in solving problems? We first performed an exploratory factor analysis (principal factor solution) with varimax rotation on the 11 activity items to see convergence among the activities. The scree test suggested two factors, which together accounted for 38.4% of the variance. Factor 1 accounted for 24.7% of the variance, and Factor 2 accounted for 13.6% of the variance. As shown in Table 3, the six activity items that were related to social capital (e.g., “assistance for building organizations and collaboration among farmers”) had high factor loadings on Factor 1 (hereafter called social capital-related activity). On the other hand, the other five activity items that were related to agricultural skills and business management (e.g., “assistance regarding food safety”, “introduction of agricultural techniques”) had high loadings on Factor 2 (hereafter called agricultural business management activity). We constructed the social capital-related activity indicator and the agricultural business management activity indicator by taking the mean of the respective items.

**Table 3:** Rotated factor matrix of the extension activity items.

	Factor loading	
Item	Social capital-related (Factor 1)	Agricultural business management (Factor 2)
Providing a vision for the future	**.57**	.09
Assistance for building organizations and collaboration among farmers	**.54**	.06
Identifying specific problems the community has	**.51**	.17
Assistance for the development of agricultural communities	**.49**	.06
Collaboration and coordination with relevant organizations	**.41**	.17
Assistance to foster the sustainable workforce	**.37**	.09
Assistance regarding food safety	.05	**.65**
Assistance to conduct eco-friendly agriculture	.05	**.64**
Assistance to establish the desirable area of productions	.27	**.35**
Introduction of agricultural techniques	.08	**.34**
Assistance for sales promotion	.24	**.32**

Factor loadings .30 or greater are shown in bold.

We then examined correlations of these two types of activity indicators with the performance score. The analysis revealed that social capital-related activity was positively related with the performance score, *r* = .39, *p*<.001. In addition, agricultural business management activity was also positively correlated with the performance score, *r* = .28, *p*<.001. As expected, however, the effect of social capital-related activity was greater than the other *t*(3899)  = 6.62, *p*<.001. This suggests that extension activities which enhance social capital are especially effective and lead to good performance to solve problems in communities.

### Analysis 2: Social capital (trust relationships) and life quality of community residents

To examine how social capital promotes the quality of life in agricultural communities, Analysis 2 focused on the effect of perceived trust relationships among the community residents (the indicator of social capital) on perceived life quality of the community residents.

First, we examined simple correlation between these two variables (Table 4). As predicted, they were found to be positively correlated with each other consistently across three datasets. This suggests that trust relationships among the community residents have a positive effect on their quality of life.

**Table 4:** Perceived trust relationships and perceived life quality of community residents (means, standard deviations, and Pearson's *r* coefficients).

	*N*	Perceived trust relationships among community residents		Perceived life quality of community residents		Correlation	
		*M*	*(SD)*	*M*	*(SD)*	*r*	*p*
Data 1	3268	5.27	(0.75)	3.83	(1.26)	.14	.000
Data 2	163	5.10	(0.65)	3.83	(1.16)	.19	.016
Data 3	97	5.36	(0.68)	4.40	(1.19)	.31	.002

*Note*. For this analysis, we included not only respondents who had data both in Data 1 and Data 2 (or Data 3) but also respondents who completed the relevant scales only in Data 2 or 3.

However, the analyses above do not indicate the causal association. To examine the causal relationships showing if perceived trust relationships really had an effect on perceived life quality, we conducted a time-series analysis by combining Data 1, 2, and 3. As mentioned above, 29 respondents in Kinki area completed the relevant scales both in Data 1 and 2. Similarly, 31 respondents in Data 3 (Aichi prefecture) were also found in Data 1, and completed the relevant scales. For both Kinki area and Aichi prefecture, the first data collection was conducted approximately one year before the second data collection. For Kinki area, responses at Time 1 were from Data 2, and responses at Time 2 came from Data 1. For Aichi prefecture, Data 1 and 3 were used for Time 1 and 2, respectively. Finally, we combined these two datasets about the two areas into single datasets to see if perceived trust relationships at Time 1 has significant effect on perceived life quality at Time 2 even after controlling for the effect of perceived life quality at Time 1.

We regressed perceived life quality at Time 2 on perceived trust relationships at Time 1, perceived life quality at Time 1, a dummy-coded variable of area (0  =  Kinki, 1  =  Aichi), and the interaction effect terms between perceived trust relationships at Time 1 and area, as well as perceived life quality at Time 1 and area (see Table 5; adjusted *R* ^2^  =  .08, *p* = .085). As expected, perceived trust relationships at Time 1 had marginally significant positive effect on perceived life quality at Time 2. Standardized regression coefficient of perceived trust relationships at Time 1 suggests that an increase of one standard deviation in this variable led to an increase of 0.34 of one standard deviation in perceived life quality measured at approximately one year later. Area did not moderate the effect of perceived trust relationships. The other effects were also found to be non-significant. In sum, the results suggest the positive effect of trust relationships on life quality of community residents.

**Table 5:** Effects of trust relationships among community residents (Time 1), life quality of community residents (Time 1), area, and their interactions on life quality of community residents (Time 2), *N* = 60.

	*b*	*(SE)*	β	*t*	*p*
Perceived trust relationships (Time 1)	0.65	(0.36)	.34	1.82	.075
Perceived life quality (Time 1)	0.29	(0.20)	.29	1.49	.142
Area (0 = Kinki, 1 = Aichi)	−0.19	(0.32)	−.08	−0.61	.543
Perceived trust relationships (Time 1) x Area	−0.61	(0.49)	−.23	−1.25	.217
Perceived life quality (Time 1) * Area	0.07	(0.26)	.05	0.28	.778

### Analysis 3: Correlates of social capital (trust relationships) of communities

As shown in Analysis 2, trust relationships among community residents, one aspect of social capital, has a positive effect on the residents' life quality. The next question is, “what enhances trust relationships among community residents?”

By analyzing Data 1, we examined correlations of perceived trust relationships among community residents with respondent's collaboration index, extraversion, communication skills, knowledge and technical skills, tie with community, and interpersonal relationships at the workplace. As shown in Table 6, collaboration index, extraversion, and communication skills were positively correlated with perceived trust relationships. Though knowledge and technical skills also had a positive association with perceived trust relationships, the effect size was quite small.

**Table 6:** Collaboration index, extraversion, communication skills, knowledge and technical skills, tie with community, and interpersonal relationships at the workplace (means, standard deviations, and Pearson's *r* coefficients with perceived trust relationships among community residents).

	*M*	*(SD)*	Correlation with perceived trust relationships among community residents	
			*r*	*p*
Respondent's personal traits				
Collaboration index	2.63	(0.34)	.17	.000
Extraversion	3.87	(1.08)	.14	.000
Communication skills	0.28	(1.41)	.17	.000
Knowledge and technical skills	−0.50	(1.58)	.08	.000
Social relationships surrounding respondent				
Tie with community	0.63	(1.15)	.34	.000
Interpersonal relationships at the workplace	1.03	(1.29)	.24	.000

*Note*. Scales ranged from 1 to 4 for Collaboration index, from 1 to 7 for Extraversion, and from −3 to 3 for the other scales.

Tie with community and interpersonal relationships at the workplace also had positive correlations with perceived trust relationships. Furthermore, these variables, which were about social relationships surrounding respondents, had positive correlations with perceived trust relationships among community residents even after controlling for the respondent's own internal traits on social relationships such as the collaboration index, extraversion, and communication skills (tie with community: *r_p_*s  = .31, .32, .30, *p*s <.001; interpersonal relationships at the workplace: *r_p_*s  = .22, .22, .21, *p*s <.001, by controlling for the collaboration index, extraversion, communication skills, respectively). These results indicate that the effects of social relationships surrounding extension officers are not spurious correlations caused by the extension officer's personal traits.

## Discussion

The concept of social capital including trust relationships and social networks has served to bring researchers' attention to the significance of social bonds for human welfare. Prior research has actually demonstrated the associations of social capital with several domains of human life, such as financial incomes, life expectancy, life satisfaction, decreased violence, maintenance of public goods, and so on [4], [9], [10].

The current study was conducted to investigate consequences and antecedents of social capital in Japanese agricultural communities by focusing on roles of professional extension officers. Extension officers are involved in many kinds of activities to help farmers, such as introducing agricultural techniques, providing managerial knowledge, and building and maintaining trust relationships and collaboration inside and around agricultural communities.

Our analyses, based on data collection including a nation-wide survey of extension officers, showed that extension activities related to social capital are particularly important. Analysis 1 revealed that to solve problems that farmers are faced with extension activities for enhancing social capital had greater effects compared to other activities such as the introduction of agricultural techniques. This finding suggests social capital plays essential roles for life in agricultural communities.

In line with the results of Analysis 1, trust relationships (one important aspect of social capital) and life quality of community residents were found to have positive association across three survey data (Analysis 2). Moreover, by analyzing panel data, we validated the causal relationship between them: Trust relationships among community residents promote their life quality.

Furthermore, to study antecedents of social capital, we explored which factors or skills of extension officers were associated with trust relationships among community residents. Analysis 3 revealed that extension officers' collaboration with related parties and communication skills were positively correlated with trust relationships within communities. In addition, interpersonal relationships at extension officers' workplace (i.e., extension centers) were positively connected with trust relationships in the local communities where the extension officers worked. This suggests a “chain effect” of social capital, meaning positive relationships in one place (extension officers' workplace) also facilitate positive relationships in another place (an agricultural community) presumably through extension officers' activities. Taken together, the current research demonstrates the importance of extension officers' work in promoting social capital in agricultural communities.

Extension activities related to agricultural techniques must not be viewed as unimportant. In fact, the demand for “specialist” function is still high. Fukuda's [27] research that collected farmers' opinions found that one of the highest-priority needs of farmers is extension of innovative techniques. Yet, the current study suggests that the other function of extension officers that have not received broad attention—“coordinator” function—has to do with a very important resource of agricultural communities, namely, social capital.

### Limitations and future directions

It is important to emphasize that our data was collected through self-report and perceptions about states of communities by extension officers. This means that the current paper relies only on the service providers' point of view, rather than the service recipients (i.e., farmers). However, it is also important to note that relying only on the farmers' point of view is not sufficient either to investigate roles of extension activities. Some extension officers we interviewed emphasized that, to motivate farmers, it is sometimes important to hide the roles of their activities from farmers. Thus, the farmers may not be aware about the functions of extension activities. It is therefore of importance to investigate associations between extension activities and communities' welfare from *both* sides. In addition to this, it would be an important future work to include objective measures, such as objective indices of farmers' health, economic success, and the actual number of cooperative interactions among farmers within communities. It is suggested that relying only on the same type of measurement (e.g., self-report likert scale) from the same source may exaggerate observed correlations due to the common method variance bias [40]. Some of our findings, however, cannot be explained solely by this bias. We found the predicted associations even when we had covariates that should share the same bias (see Analyses 2 and 3). Yet, it is undoubtedly desirable to have objective measures as well and to examine the robustness of the findings.

Another limitation of the current study is the small sample size of the panel data for Analysis 2. We needed to include only respondents who had worked for the same community for both Time 1 and Time 2 in the analysis. We scarcely had a fair amount of respondents since extension officers' working terms for one community are generally short (in Data 1, we asked the respondents how long they had been working for the same community; the length of the mean time was 2.18 years, median was 1.50 years, and mode was 0.50 years). Future studies collecting panel data through those who stay in the same community for a longer period of time (e.g., farmers) are needed. It is also important to point out that our finding from the time-series analysis (Analysis 2) is not conclusive about the causality. By controlling for the effect of life quality at Time 1, we could show that the opposite causality (i.e., life quality promotes trust relationships among community residents) cannot fully explained the observed association. However, it is still possible that a third variable explains the association between trust relationships and life quality. For example, it may be possible that existence of strong leadership in a community promotes both trust relationships among residents and their life quality. Future studies that examine effects of such potential third variables are needed.

Additionally, it is also important to investigate potential negative effects of social capital on welfare of agricultural communities. For example, it has been suggested that excessive levels of bonding-type social capital (social capitals within a group) may promote distrust toward outsiders and inhibit the group's economic growth [41]. Future studies need to investigate what kinds of extension activities promote (or inhibit) social networks crossing a boundary of local communities.

There is another important question that future research needs to address: How can we train good coordinators? Though it is suggested by the current study that good coordinators (e.g., extension officers who have high communication skills) can help communities enhance trust relationships and collaborations, knowledge on how to foster such good coordinators is requisite to keep communities benefiting from them in the future. Thus, we need to know, for example, how to obtain ability for collaboration, how to acquire good communication skills, and how to recruit those who are (or have potential to be) good coordinators. Also, though the current study targeted extension officers and social capital in Japanese agricultural communities, presumably other types of communities, organizations, and groups face similar problems and thus coordinators may play crucial roles. How to achieve efficient problem solving in groups is one of the questions social psychological research has extensively addressed. From the findings of the current study, skilled coordinators are expected to play significant roles in groups and organizations that need cooperation and collaboration among members, such as medical institutions and educational institutions. Future research is needed to find ways to build systems that can sustainably provide coordinators who support building connections between people.
